# Cost-effectiveness of healthy eating and/or physical activity promotion in pregnant women at increased risk of gestational diabetes mellitus: economic evaluation alongside the DALI study, a European multicenter randomized controlled trial

**DOI:** 10.1186/s12966-018-0643-y

**Published:** 2018-03-14

**Authors:** Karen Broekhuizen, David Simmons, Roland Devlieger, André van Assche, Goele Jans, Sander Galjaard, Rosa Corcoy, Juan M. Adelantado, Fidelma Dunne, Gernot Desoye, Jürgen Harreiter, Alexandra Kautzky-Willer, Peter Damm, Elisabeth R. Mathiesen, Dorte M. Jensen, Liselotte L. Andersen, Annunziata Lapolla, Maria G. Dalfra, Alessandra Bertolotto, Ewa Wender-Ozegowska, Agnieszka Zawiejska, David Hill, Frank J. Snoek, Judith G. M. Jelsma, Judith E. Bosmans, Mireille N. M. van Poppel, Johanna M. van Dongen

**Affiliations:** 1 Department of Health Sciences and EMGO+ Institute for Health and Care Research, Vrije Universiteit Amsterdam, Amsterdam, The Netherlands; 2 Western Sydney University, Campbelltown, NSW Australia; 3 Institute of Metabolic Science, Addenbrooke’s Hospital, Cambridge, England; 4 Department of Development and Regeneration: Pregnancy, Fetus and Neonate, Gynaecology and Obstetrics, University Hospitals, Katholieke Universiteit Leuven, Leuven, Belgium; 5 Department of Obstetrics and Gynaecology, Division of Obstetrics and Prenatal Medicine, Erasmus MC, University Medical Centre Rotterdam, Rotterdam, The Netherlands; 6 Institut de Recerca de l’Hospital de la Santa Creu i Sant Pau, Barcelona, Spain; 7 CIBER Bioengineering, Biomaterials and Nanotechnology, Instituto de Salud Carlos III, Zaragoza, Spain; 8 Galway Diabetes Research Centre and College of Medicine Nursing and Health Sciences, National University of Ireland, Galway, Ireland; 9 Department of Obstetrics and Gynecology, Medizinische Universitaet Graz, Graz, Austria; 10 Gender Medicine Unit, Endocrinology and Metabolism, Dept. Internal Medicine III, Medical University of Vienna, 1090 Vienna, Austria; 11 Center for Pregnant Women with Diabetes, Departments of Endocrinology and Obstetrics, Rigshospitalet, Institute of Clinical Medicine, Faculty of Health and Medical Sciences, University of Copenhagen, Copenhagen, Denmark; 12 Department of Endocrinology, Odense University Hospital, Odense, Denmark; 13 Department of Gynecology and Obstetrics, Odense University Hospital, Odense, Denmark; 14 Department of Clinical Research, Faculty of Health Sciences, University of Southern Denmark, Odense, Denmark; 15 Universita Degli Studi di Padova, Padua, Italy; 16 Azienda Ospedaliero Universitaria – Pisa, Pisa, Italy; 17 Medical Faculty, Poznan University of Medical Sciences, Poznan, Poland; 18 Recherche en Santé Lawson SA, Bronschhofen, Switzerland; 19 Department of Medical Psychology, EMGO+-Institute for Health and Care Research, VU University Medical Centre, Amsterdam, The Netherlands; 20 Department of Medical Psychology, Academic Medical Centre, Amsterdam, The Netherlands; 21 Department of Public and Occupational Health, EMGO+ Institute for Health and Care Research, VU University Medical Centre, 1081 BT Amsterdam, The Netherlands; 22 Institute of Sport Science, University of Graz, 8010 Graz, Austria; 23 Department of Health Sciences and EMGO+ Institute for Health and Care Research, Faculty of Earth & Life Sciences, VU University Amsterdam, De Boelelaan 1085, 1081 HV Amsterdam, The Netherlands

**Keywords:** Economic evaluation, Cost-effectiveness, Gestational diabetes, Lifestyle intervention, Pregnant women

## Abstract

**Background:**

Gestational diabetes mellitus (GDM) is associated with perinatal health risks to both mother and offspring, and represents a large economic burden. The DALI study is a multicenter randomized controlled trial, undertaken to add to the knowledge base on the effectiveness of interventions for pregnant women at increased risk for GDM. The purpose of this study was to evaluate the cost-effectiveness of the healthy eating and/or physical activity promotion intervention compared to usual care among pregnant women at increased risk of GDM from a societal perspective.

**Methods:**

An economic evaluation was performed alongside a European multicenter-randomized controlled trial. A total of 435 pregnant women at increased risk of GDM in primary and secondary care settings in nine European countries, were recruited and randomly allocated to a healthy eating and physical activity promotion intervention (HE + PA intervention), a healthy eating promotion intervention (HE intervention), or a physical activity promotion intervention (PA intervention). Main outcome measures were gestational weight gain, fasting glucose, insulin resistance (HOMA-IR), quality adjusted life years (QALYs), and societal costs.

**Results:**

Between-group total cost and effect differences were not significant, besides significantly less gestational weight gain in the HE + PA group compared with the usual care group at 35–37 weeks (−2.3;95%CI:-3.7;-0.9). Cost-effectiveness acceptability curves indicated that the HE + PA intervention was the preferred intervention strategy. At 35–37 weeks, it depends on the decision-makers’ willingness to pay per kilogram reduction in gestational weight gain whether the HE + PA intervention is cost-effective for gestational weight gain, whereas it was not cost-effective for fasting glucose and HOMA-IR. After delivery, the HE + PA intervention was cost-effective for QALYs, which was predominantly caused by a large reduction in delivery-related costs.

**Conclusions:**

Healthy eating and physical activity promotion was found to be the preferred strategy for limiting gestational weight gain. As this intervention was cost-effective for QALYs after delivery, this study lends support for broad implementation.

**Trial registration:**

ISRCTN ISRCTN70595832. Registered 2 December 2011.

**Electronic supplementary material:**

The online version of this article (10.1186/s12966-018-0643-y) contains supplementary material, which is available to authorized users.

## Background

Glucose intolerance with its onset during pregnancy (i.e. Gestation Diabetes Mellitus; GDM) is associated with perinatal health risks to both mother and offspring, and represents a large economic burden [1–4]. Women experiencing GDM are also at increased risk for the future development of type-2 diabetes, where prevention through lifestyle change has been found to be cost-effective among high risk individuals [5–8]. As many of the pathophysiological processes underlying GDM are similar to those of type-2 diabetes, such interventions could also be useful for GDM prevention. However, to avoid fetal harm, they should aim to limit gestational weight gain, rather than reduce weight [9].

Evidence on the effectiveness of interventions aimed at limiting gestational weight gain is mixed. A meta-analysis found dietary counseling to significantly reduce GDM, whereas no effect on maternal fasting glucose was found [10]. A more recent systematic review of 13 randomized controlled trials did not find a significant difference in the risk of developing GDM between women receiving a physical activity and healthy eating promotion intervention compared with those receiving no intervention [11]. However, as the methodological quality of the existing evidence is low to moderate, more high quality randomized controlled trials are needed to investigate the effectiveness of interventions that are aimed at pregnant women at increased risk of GDM.

To add to the knowledge base on the effectiveness of interventions for pregnant women at increased risk for GDM, the DALI study was undertaken. The DALI study is a multicenter randomized controlled trial conducted in nine European countries [12]. The study found a healthy eating and physical activity promotion intervention to reduce gestational weight gain, but not GDM risk [13]. There is evidence, however, that limiting gestational weight gain could be beneficial in its own right through improved obstetric outcomes [14, 15].

Decisions about investments in health programs are not only guided by their effectiveness, but also by their additional costs in relation to these effects (i.e. cost-effectiveness) [16]. The latter is evaluated through an economic evaluation. In times of increasing healthcare costs and tight budgets, such studies provide important information for decision-makers to weigh alternative courses of action and to decide which programs to implement and/or reimburse [17].

The present study aimed to evaluate the cost-effectiveness of a healthy eating and/or physical activity promotion intervention compared to usual care among pregnant women at increased risk of GDM from a societal perspective.

## Methods

### Design and participants

The DALI study is a multicenter randomized controlled trial with a factorial study design that was conducted in nine European countries (2012–2015) [12, 13]. Pregnant women attending a participating antenatal clinic or hospital were asked to participate in the study. The participating centers were located in the United Kingdom, Ireland, Austria, Poland, Italy (Padua, Pisa), Spain, Denmark (Odense, Copenhagen), Belgium, and the Netherlands. Pregnant women with a pre-pregnancy body mass index (BMI) of ≥29 kg/m^2^ were eligible for inclusion [12]. Further inclusion criteria were ≤19 + 6 days of gestation, having a singleton pregnancy, and being aged ≥18 years. Women were excluded if they: were diagnosed with GDM by oral glucose tolerance testing [18]; had pre-existing diabetes; were not able to walk ≥100 m safely; required complex diets; had chronic medical conditions; had a psychiatric disorder; were not fluent in the major language of the country of recruitment or were unable to have a conversation with the lifestyle coach in another language for which translated intervention materials were available. After the provision of informed consent, baseline assessment occurred <20 weeks of gestation and was immediately followed by randomization [12]. The CONSORT (Consolidated Standards Of Reporting Trials) checklist is available as Additional file 1.

### Randomization

**Fig. 1:**
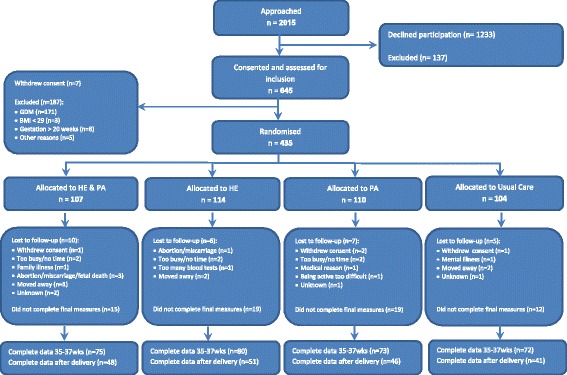
CONSORT diagram. CONSORT diagram of recruitment, randomization and drop out of the DALI lifestyle trial

Participants were randomly allocated to one of the four arms of the DALI study, comparing 1) a healthy eating and physical activity promotion intervention (HE + PA intervention), 2) a healthy eating promotion intervention (HE intervention), and 3) a physical activity promotion intervention (PA intervention) with 4) usual care (Fig. 1). Randomization was performed using a computerized random number generator and was pre-stratified for intervention center and the trial’s 2 × 2 design. The trial coordinator prepared and distributed sealed opaque envelopes containing the intervention arm to which the participants were allocated. Prior to the start of the intervention, the allocation outcome was communicated to the participants by the lifestyle coach.

### DALI intervention

The DALI lifestyle coaching intervention was targeted at HE + PA, HE, or PA. A detailed description of the intervention conditions is reported elsewhere [12] .In brief, lifestyle coaching was offered during five face-to-face sessions of 30–45 min and four optional telephone calls of ≤20 min that occurred between the face-to-face sessions. Participants were assigned to one lifestyle coach [19, 20]. Face-to-face sessions occurred at the participants’ home or at the nearest hospital, midwife practice, or general practice. To optimize intervention uptake, timing and intervals between face-to-face and telephone contacts were tailored to the participants’ preferences. It was stressed, however, that at least 4 face-to-face coaching sessions should occur before the second measurement and that the intervention should be finished before 35 weeks of gestation. Lifestyle coaches used a PDA (or, where unavailable, a paper version), providing a framework for the face-to-face and telephone sessions and guidance for intervention delivery. Detailed description of the DALI intervention is reported in the TIDieR (Template for Intervention Description and Replication) checklist in Additional file 2.

### Usual care

During pregnancy, usual care group participants received care as usual by their midwife or obstetrician and did not receive any of the lifestyle interventions.

### Baseline characteristics

Potential confounders were assessed at baseline by questionnaire, including age (years), ethnicity (European descent:yes/no), multiparous pregnancy (yes/no), education level (higher education:yes/no), history of GDM (yes/no), height (cm), pre-pregnancy weight and weight at study entry (kilogram), BMI (kg/m^2^), and gestation at study entry (weeks).

### Study outcomes

Clinical outcomes included gestational weight gain, fasting glucose, insulin resistance (HOMA-IR), and health-related quality of life [12, 13].

Weight was measured at baseline (<20 weeks), 24–28 weeks, and 35–37 weeks. Gestational weight gain was defined as the participants’ weight change from baseline to 35–37 weeks. Fasting glucose and HOMA-IR were measured at baseline, 24–28 weeks, and 35–37 weeks as well. Anthropometric and laboratory measurements followed a standardized methodology as described elsewhere [12].

Health-related quality of life was assessed at baseline (<20 weeks), 24–28 weeks, and at 35–37 weeks using the EQ-5D-3 L [21]. Since many costs that are associated with the interventions under study are likely related to the delivery of babies, costs were measured until after delivery (i.e. 24–28 h after delivery). Therefore, health-related quality of life was assessed at 24–48 h after delivery as well. Utilities were estimated using the European tariff [22]. Quality adjusted life years (QALYs) were calculated by multiplying the utilities by the amount of time spent in a health state. For this purpose, transitions between EQ-5D-3 L health states were linearly interpolated.

### Cost measures

Resource use data were collected by questionnaire at 24–28 weeks, 35–37 weeks, and 24–48 after delivery. Additional resource use data concerning the delivery and other delivery-related in-hospital services were collected from hospital records. Costs were measured from a societal perspective and included intervention, medical, absenteeism, and travel costs. All costs were expressed in Euros 2012 [23]. Due to a limited availability of unit costs reflecting the “true cost” of a good or service for most of the participating countries, unit costs were based on Dutch costing data. To correct for the fact that the relative prices of factors differ across countries, unit costs were adjusted per country using purchasing power parities [24, 25]. An overview of the unit costs per country can be found in Additional file 3.

Intervention costs included all costs related to the development, implementation, and execution of the interventions, and were estimated using a bottom-up micro-costing approach. The latter means that intervention costs were estimated by collecting detailed information regarding the quantities of resources consumed while implementing and executing the interventions as well as their respective unit prices. Frequency and duration of face-to-face and telephone sessions were based on pilot study data [13]. Time investments of intervention providers were valued using their gross hourly salaries. Material and website hosting costs were estimated using invoices. Medical costs included costs related to the use of primary healthcare (i.e. care by a GP, a midwife, a dietician, and a diabetes counselor), secondary healthcare (i.e. outpatient hospital visits, maternal hospitalization, neonatal hospitalization, delivery, and in-hospital ultrasounds, fetal non-stress tests, and blood tests) and medication. Primary and secondary healthcare use were valued using Dutch standard costs [26]. If unavailable, prices according to professional organizations, hospital records, or a previously published economic evaluation were used [27]. Medication use was valued using prices of the *“Royal Dutch Society of Pharmacy”* [28]*.* Absenteeism costs were estimated according to the human capital approach, using the estimated price of productivity losses per sickness absence day based on 5-year age categories [26]. Unit costs for valuing travel costs were derived from the *“Dutch Manual of costing”* and varied by mode of transportation [26].

### Statistical analysis

Analyses were performed according to the intention-to-treat principle. Missing data were replaced using multiple imputation, stratified by treatment group. The imputation model included variables related to the “missingness” of data or the outcome measure, and variables that differed at baseline between groups [29]. Using Predictive Mean Matching, 20 complete data sets were created to keep the loss-of-efficiency below 5% [29]. Datasets were analyzed separately as specified below. Pooled estimates were calculated using Rubin’s rules [30].

Effects on gestational weight gain, fasting glucose, and HOMA-IR at 35–37 weeks and the effect on QALYs gained from baseline till after delivery were analyzed using multilevel analyses, adjusted for baseline values and follow-up duration (in weeks). Two levels were identified: study centers (*n* = 11) and participants (*n* = 435). Total and disaggregated cost differences were also estimated using linear multilevel analyses, with the same two-level structure and adjusted for follow-up duration as well. 95%CIs around cost differences were estimated using bias-corrected (BC) bootstrapping, with 5000 replications (stratified by study center). Incremental cost-effectiveness ratios (ICERs) were calculated by dividing the total cost differences by those in effects. For this purpose, the difference in total costs at 35–37 weeks was divided by the difference in gestational weight gain, fasting glucose, and HOMA-IR at 35–37 weeks and the difference in total costs after delivery by the difference in QALYs gained after delivery. Bootstrapped incremental cost-effect pairs were plotted on cost-effectiveness planes. Cost-effectiveness acceptability curves (CEACs) were constructed, showing the interventions’ probability of being cost-effective compared with usual care at various willingness-to-pay (WTP) values.

Analyses were performed using Stata 12.0. Statistical significance was set at *p* < 0.05.

### Sensitivity analyses

Four sensitivity analyses (SAs) were performed. In SA1, national tariffs were used for estimating QALYs [31–35], instead of the European tariff. In SA2, United Kingdom (UK) unit costs data were used for valuing resource use, instead of Dutch ones. UK unit costs were used in SA2, as previous research indicates that a large share of European economic evaluations is carried out in the UK, and thus relies on UK costing data as well [36]. UK unit costs were derived from previously published studies, international databases, hospital records, and published UK pricing lists [37–40]. In SA3, analyses were performed from the healthcare perspective. In SA4, solely participants with complete cost and effect data were included.

## Results

### Participants

Of the participants, 107 were randomized to the HE + PA group, 114 to the HE group, 110 to the PA group, and 104 to the usual care group (Fig. 1). At baseline, demographic and clinical characteristics were comparable across group (Table 1). After 35–37 weeks, complete data were available for 75 participants (68%) in the HE + PA group, 80 participants (70%) in the HE group, 73 participants (68%) in the PA group, and 72 participants (69%) in the usual care group. After delivery, complete data were available for 48 participants (44%) in the HE + PA group, 51 participants (45%) in the HE group, 46 participants (43%) in the PA group, and 41 participants (39%) in the usual care group. Resource use data concerning the delivery of babies and other delivery-related in-hospital services were complete for the majority of participants (*n* = 359; 83%). Relevant differences were found between participants with complete and incomplete data in terms of the country they lived in, and their ethnicity, pre-pregnancy weight, and marital status (Table 1).

**Table 1:** Baseline characteristics of the participants

Variable	Usual Care *N* = 104	Complete *N* = 41	Incomplete *N* = 63	HE + PA *N* = 107	Complete *N* = 48	Incomplete *N* = 59	HE *N* = 114	Complete *N* = 51	Incomplete N = 63	PA *N* = 110	Complete *N* = 46	Incomplete *N* = 64
Age, y, mean ± SD	31.8 ± 5.6	32.1 ± 6.1	31.5 ± 5.2	31.9 ± 5.3	33.3 ± 4.7	30.7 ± 5.4	32.5 ± 5.5	32.9 ± 4.7	32.1 ± 6.1	31.7 ± 5.1	33.0 ± 4.4	30.7 ± 5.4
Country N (%)												
Netherlands	8 (8%)	3 (7%)	5 (8%)	10 (9%)	8 (17%)	2 (3%)	8 (7%)	3 (6%)	5 (8%)	9 (8%)	7 (15%)	2 (3%)
Belgium	7 (6%)	2 (5%)	5 (8%)	11 (10%)	4 (8%)	7 (12%)	11 (10%)	4 (8%)	7 (11%)	10 (9%)	2 (4%)	8 (13%)
United Kingdom	11 (11%)	4 (10%)	7 (11%)	10 (9%)	5 (10%)	5 (8%)	13 (11%)	6 (12%)	7 (11%)	11 (10%)	4 (9%)	7 (11%)
Denmark	27 (26%)	9 (22%)	18 (29%)	25 (23%)	6 (13%)	19 (32%)	25 (22%)	7 (14%)	18 (29%)	25 (23%)	4 (9%)	21 (33%)
Italy	14 (13%)	3 (7%)	11 (17%)	13 (12%)	7 (15%)	6 (10%)	14 (12%)	8 (16%)	6 (10%)	15 (14%)	8 (17%)	7 (11%)
Spain	9 (9%)	7 (17%)	1 (2%)	10 (9%)	7 (15%)	3 (5%)	10 (9%)	6 (12%)	4 (6%)	9 (8%)	7 (15%)	2 (3%)
Ireland	10 (10%)	2 (5%)	8 (13%)	10 (9%)	3 (6%)	7 (12%)	12 (11%)	3 (6%)	9 (14%)	13 (12%)	5 (11%)	8 (13%)
Poland	10 (10%)	7 (17%)	3 (5%)	7 (7%)	4 (8%)	3 (5%)	10 (9%)	6 (12%)	4 (6%)	11 (10%)	5 (11%)	6 (9%)
Austria	11 (11%)	5 (12%)	6 (19%)	11 (10%)	4 (8%)	2 (3%)	11 (10%)	8 (16%)	3 (5%)	9 (8%)	4 (9%)	5 (8%)
Having a partner N (%)	99 (95%)	38 (93%)	61 (97%)	99 (93%)	47 (98%)	52 (88%)	109 (96%)	50 (98%)	59 (94%)	103 (94%)	43 (93%)	60 (94%)
Multiparous, N (%)	49 (47%)	16 (39%)	33 (52%)	56 (52%)	25 (52%)	31 (53%)	65 (57%)	33 (65%)	32 (51%)	51 (46%)	26 (57%)	25 (39%)
European descent, N (%)	93 (89%)	34 (83%)	59 (94%)	94 (88%)	42 (88%)	52 (88%)	96 (84%)	45 (88%)	51 (81%)	94 (86%)	37 (80%)	57 (89%)
Higher education, N (%)	53 (52%)	21 (51%)	33 (53%)	58 (54%)	29 (60%)	29 (49%)	65 (57%)	29 (57%)	36 (57%)	60 (55%)	26 (57%)	34 (53%)
History of GDM, N (%)	3 (3%)	1 (2%)	2 (3%)	4 (4%)	2 (4%)	2 (3%)	7 (6%)	4 (8%)	3 (5%)	4 (4%)	1 (2%)	3 (5%)
Gestation on entry, weeks, mean ± SD	15.2 ± 2.4	14.9 ± 2.6	15.3 ± 2.1	15.2 ± 2.2	15.1 ± 2.1	15.3 ± 2.3	15.3 ± 2.4	15.4 ± 2.5	15.3 ± 2.4	15.5 ± 2.3	15.0 ± 2.6	15.9 ± 2.0
Pre-pregnancy weight, kg, mean ± SD	92.0 ± 11.5	91.1 ± 11.8	92.6 ± 11.4	93.3 ± 13.7	91.4 ± 14.6	94.7 ± 12.9	92.5 ± 13.6	89.9 ± 11.1	95.5 ± 15.1	92.7 ± 13.4	92.1 ± 13.8	93.1 ± 13.1
Weight at entry, kg, mean ± SD	94.2 ± 12.6	93.0 ± 12.0	94.9 ± 13.0	95.2 ± 13.8	93.8 ± 15.4	96.2 ± 12.3	94.8 ± 13.2	92.1 ± 10.8	97.0 ± 14.5	94.6 ± 12.8	94.1 ± 90.1	95.1 ± 12.5
Height, cm, mean ± SD	165.9 ± 6.7	165.7 ± 6.9	165.9 ± 6.6	166.0 ± 6.6	165.4 ± 7.3	166.4 ± 6.1	165.1 ± 6.6	163.9 ± 6.7	166.1 ± 6.4	165.6 ± 7.2	165.5 ± 6.7	165.7 ± 7.5
BMI at entry, kg/m^2^, mean ± SD	34.2 ± 3.9	34.8 ± 2.8	34.5 ± 4.4	34.5 ± 4.0	34.2 ± 3.9	34.7 ± 4.0	34.7 ± 4.2	34.3 ± 3.5	35.1 ± 4.7	34.4 ± 3.8	34.3 ± 3.9	34.6 ± 3.7
Fasting glucose, mmol/l, mean ± SD	4.7 ± 0.4	4.7 ± 0.4	4.7 ± 0.3	4.6 ± 0.3	4.7 ± 0.3	4.5 ± 0.3	4.6 ± 0.4	4.7 ± 0.4	4.5 ± 0.4	4.6 ± 0.4	4.5 ± 0.4	4.6 ± 0.4
HOMA-IR, mean ± SD	1.0 ± 0.6	1.1 ± 0.5	1.0 ± 0.6	1.0 ± 0.4	0.9 ± 0.4	1.0 ± 0.4	0.9 ± 0.5	0.9 ± 0.5	0.9 ± 0.4	0.9 ± 0.4	0.9 ± 0.4	1.0 ± 0.4
Utility value, mean ± SD	0.86 ± 0.02	0.87 ± 0.03	0.85 ± 0.02	0.89 ± 0.01	0.90 ± 0.02	0.88 ± 0.02	0.86 ± 0.01	0.86 ± 0.02	0.87 ± 0.02	0.85 ± 0.02	0.84 ± 0.03	0.86 ± 0.02

Abbreviations: N: Number; SD: Standard Deviation; GDM: Gestational Diabetes Mellitus, kg: kilogram; m: meter; cm: centimetre; BMI; Body Mass Index; mmol/l; millimol per liter; HOMA-IR; HOMA index – Insulin Resistence

### Effectiveness

After 35–37 weeks, gestational weight gain (in kilograms) was significantly lower in the HE + PA group compared with the usual care group (−2.3;95%CI:-3.7 to −0.9). Gestational weight gain was also lower in the HE group compared with the usual care group, but this difference was not significant. There was no significant beneficial effect on fasting glucose, HOMA-IR, and QALYs (Table 2).

**Table 2:** Cost-effectiveness analysis results (main analysis – Societal perspective)

HE + PA									
Outcome measure	Sample size		∆C (95%CI)	∆E (95%CI)	ICER	Distribution CE-plane (%)			
	Intervention	Control	€	Points	€/point	NE	SE	SW	NW
Gestational weight gain	107	104	380 (−811 to 1510)	−2.3 (−3.7 to −0.9)	−165	73.6	26.4	0.0	0.0
Fasting glucose	107	104	380 (−811 to 1510)	0.0 (−0.2 to 0.1)	−9198	52.9	20.2	6.3	20.7
HOMA-IR	107	104	380 (−811 to 1510)	0.0 (−0.1 to 0.2)	8971	47.4	16.2	10.1	26.2
QALYs	107	104	−1627 (−4000 to 556)	0.02 (0.00 to 0.04)	−91,254	7.8	88.8	3.0	0.4
HE									
Outcome measure	Sample size		∆C (95%CI)	∆E (95%CI)	ICER	Distribution CE-plane (%)			
	Intervention	Control	€	Points	€/point	NE	SE	SW	NW
Gestational weight gain	114	104	648 (−482 to 1759)	−0.6 (−2.4 to 1.2)	−1058	66.1	9.8	3.5	20.5
Fasting glucose	114	104	648 (−482 to 1759)	0.1 (0.0 to 0.3)	5247	2.7	0.7	12.6	83.9
HOMA-IR	114	104	648 (−482 to 1759)	0.2 (0.0 to 0.3)	4302	80.5	12.2	1.2	6.2
QALYs	114	104	653 (−1997 to 3343)	0.00 (−0.02 to 0.02)	−241,959	24.9	12.9	18.7	43.6
PA									
Outcome measure	Sample size		∆C (95%CI)	∆E (95%CI)	ICER	Distribution CE-plane (%)			
	Intervention	Control	€	Points	€/point	NE	SE	SW	NW
Gestational weight gain	110	104	710 (−486 to 1875)	0.2 (−1.4 to 1.7)	4810	34.4	7.6	7.1	50.9
Fasting glucose	110	104	710 (−486 to 1875)	0.0 (−0.1 to 0.1)	−74,480	48.8	8.9	5.8	36.6
HOMA-IR	110	104	710 (−486 to 1875)	0.1 (−0.1 to 0.3)	11,292	61.7	10.6	4.1	22.7
QALYs	110	104	−1155 (−3473 to 1142)	0.00 (−0.03 to 0.01)	146,179	2.9	16.3	65.9	14.8

*Abbreviations*: *C* Costs, *E* Effects, *ICER* Incremental Cost-Effectiveness Ratio, *CE-plane* Cost-Effectiveness plane, *NE* Northeast-Quadrant, *SE* Southeast-Quadrant, *NW* Northwest-Quadrant, *ZW* Southwest-Quadrant

### Costs

Average intervention costs per participant ranged from €426 (SEM = 8) in the PA group to €436 (SEM = 7) in the HE + PA group. Table 3 provides an overview of all total and disaggregate cost differences. At 35–37 weeks, no significant differences in total societal costs were found, but primary healthcare costs were significantly lower in the HE + PA group compared with the usual care group (−39;95%CI:-75 to −3). After delivery, total societal costs were lower in the HE + PA group and the PA group than in the usual care group and higher in the HE group than in the usual care group, but these between-group differences were not significant. Costs related to the delivery of the babies were the biggest contributor to the total cost differences (Table 3).

**Table 3:** Mean cost per participant and adjusted mean cost differences (main analysis – Societal perspective)

Cost category	Usual Care (*n* = 104); mean (SEM)	HE + PA (*n* = 110); mean (SEM)	HE + PA versus Usual Care ∆C (95%CI)	HE (*n* = 114); mean (SEM)	HE versus Usual Care ∆C (95%CI)	PA (*n* = 107); mean (SEM)	PA versus Usual Care ∆C (95%CI)
35–37 weeks							
Intervention	0 (0)	436 (7)	436 (416 to 455)	430 (8)	430 (411 to 449)	426 (8)	426 (407 to 445)
Medical	475 (59)	530 (97)	62 (−142 to 296)	398 (47)	−66 (−225 to 71)	382 (32)	−83 (−222 to 37)
Primary healthcare	180 (15)	141 (15)	−39 (−75 to −3)	148 (13)	−25 (−56 to 5)	148 (12)	−28 (−60 to 4)
Secondary healthcare	159 (20)	155 (24)	−1 (−56 to 55)	158 (21)	4 (−50 to 58)	173 (21)	14 (−39 to 68)
Medication	137 (45)	234 (83)	99 (−70 to 306)	91 (34)	−42 (−163 to 60)	61 (15)	−71 (−182 to 8)
Absenteeism	2235 (484)	2032 (415)	−102 (−1260 to 975)	2511 (446)	264 (−847 to 1341)	2608 (533)	386 (−808 to 1539)
Travel	26 (3)	22 (4)	−3 (−10 to 5)	21 (2)	−4 (−10 to 2)	27 (5)	1 (−7 to 11)
TOTAL	2736 (494)	3020 (439)	380 (−811 to 1510)	3361 (450)	648 (−482 to 1759)	3444 (539)	710 (−486 to 1875)
Cost category	Control (*n* = 104); mean (SEM)	HE + PA (*n* = 110); mean (SEM)	∆C (95%CI)	HE (*n* = 114); mean (SEM)	∆C (95%CI)	PA (*n* = 107); mean (SEM)	∆C (95%CI)
After delivery							
Intervention	0 (0)	436 (7)	436 (416 to 455)	430 (8)	430 (411 to 449)	426 (8)	426 (407 to 445)
Medical	7646 (737)	5983 (513)	−1490 (−3164 to 129)	6986 (902)	−504 (−2386 to 1642)	5189 (416)	−2286 (−3386 to −841)
Primary healthcare	235 (18)	197 (19)	−42 (−86 to 0)	202 (15)	−20 (−61 to 24)	202 (15)	−31 (−72 to 8)
Delivery-related	6962 (713)	5165 (466)	−1614 (−3201 to 109)	6334 (898)	−471 (−2290 to 1645)	4552 (393)	−2242 (−3779 to −871)
Secondary healthcare - other	262 (24)	240 (32)	−23 (−93 to 54)	266 (28)	9 (−59 to 80)	284 (32)	22 (−49 to 100)
Medication	187 (67)	381 (125)	188 (−58 to 490)	173 (53)	−18 (−198 to 135)	151 (44)	−36 (−202 to 103)
Absenteeism	3567 (729)	2921 (522)	−567 (−2194 to 955)	4295 (763)	676 (−1048 to 2429)	5252 (825)	709 (−1102 to 2532)
Travel	36 (3)	34 (5)	−3 (−12 to 9)	37 (5)	1 (−8 to 12)	39 (6)	2 (−8 to 15)
TOTAL	11,249 (1035)	9374 (725)	−1627 (−4000 to 556)	11,749 (1172)	653 (−1997 to 3343)	9907 (885)	−1155 (−3473 to 1142)

*Abbreviations*: *n* Number, *SEM* Standard Error of the Mean, *C* Costs

### Cost-effectiveness: Societal perspective

**Fig. 2:**
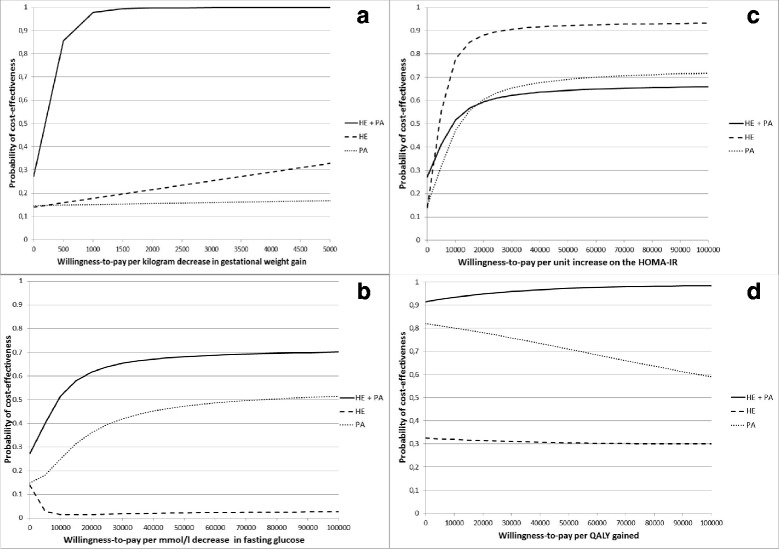
Cost-effectiveness acceptability curves. Cost-effectiveness acceptability curves showing the probabilities of the intervention’s being cost-effective in comparison with usual care for gestational weight gain (**a**), fasting glucose (**b**), and HOMA-IR (**c**), and QALYs (**d**)

For gestational weight gain, ICERs indicated that the HE + PA intervention and the HE intervention were on average *more costly* and *more effective* than usual care, while the PA intervention was on average *more costly* and *less effective* than usual care (Table 2). Cost-effectiveness acceptability curves indicated that if decision-makers are not willing to pay anything per kilogram decrease in gestational weight gain (i.e. willingness-to-pay [WTP] = €0/kg), the likelihood of the intervention being cost-effectiveness compared to usual care was low for all interventions (i.e. a probability ≤0.27). At all WTP values, the HE + PA intervention had the highest likelihood of being cost-effective in comparison with usual care. Given a WTP value of €600/kg and €750/kg, for example, this intervention was 90% and 95% more likely to be cost-effective than usual care, while the likelihood of the HE intervention or the PA intervention being more cost-effective than usual care was much lower (Fig. 2a).

For fasting glucose, ICERs indicated that the HE + PA intervention and the PA intervention were on average *more costly* and *more effective* than usual care, while the HE intervention was on average *more costly* and *less effective* than usual care (Table 2). The likelihood of the interventions’ being cost-effective compared with usual care was low if decision-makers are not willing to pay anything per mmol/l decrease in fasting glucose (i.e. a probability ≤0.27). At higher WTP values, the HE + PA and PA intervention’s likelihood of being cost-effective in comparison with usual care gradually increased, while that of the HE intervention gradually decreased. The HE + PA intervention reached the highest likelihood of being cost-effective compared with usual care (i.e. a 0.72 probability at a WTP of €600,000/mmol/l)(Fig. 2b).

For HOMA-IR, ICERs indicated that all interventions were on average *more costly* and *more effective* than usual care (Table 2). Again, the likelihood of the interventions’ being cost-effective compared with usual care was low if decision-makers are not willing to pay anything per unit increase on the HOMA-IR (i.e. a probability ≤0.27). At higher WTP values, all of the interventions’ likelihood of being cost-effective compared with usual care increased, with the HE intervention reaching the highest likelihood of being cost-effective compared with usual care (i.e. a 0.93 probability at a WTP of €100,000/unit)(Fig. 2c).

For QALYs, ICERs indicated that the HE + PA intervention was on average *less costly* and *more effective* than usual care, the HE intervention was on average *more costly* and *less effective* than usual care, and the PA intervention was on average *less costly* and *less effective* than usual care (Table 2). If decision-makers are not willing to pay anything per QALY gained, the HE + PA intervention and the PA intervention had a relatively high likelihood of being cost-effective compared with usual care (i.e. 0.91 for the HE + PA intervention and 0.82 for the PA intervention), while that of the HE intervention was low (i.e. 0.32). At all WTP values, the HE + PA intervention had the highest likelihood of being cost-effective in comparison with usual care. Given a WTP value of €10,000/QALY and €80,000/QALY, for example, this intervention was 93% and 98% more likely to be cost-effective than usual care (Fig. 2d).

### Sensitivity analyses

Sensitivity analyses indicated that the overall conclusions of the current study would not change when using national QALY tariffs instead of European ones (SA1) and UK unit cost data instead of Dutch unit cost data (SA2). However, when the healthcare perspective was applied instead of the societal perspective (SA3), total costs until after delivery were significantly lower in the PA group than in the usual care group, whereas this difference was not significant in the main analysis. As a consequence, the PA intervention had the highest likelihood of being cost-effective compared with usual care if decision-makers are not willing to pay anything per QALY gained, whereas in the main analysis this was the case for the HE + PA intervention. In accordance with the results of the main analysis, however, the HE + PA intervention had the highest likelihood of being cost-effective compared with usual care at WTP values of €35,000/QALY or more. When only participants with complete data were included in the analysis instead of all participants (SA4), the HE + PA intervention and PA intervention had higher total costs compared with usual care after delivery, whereas they were lower in the main analysis. In both analyses, however, these differences in costs were not significant. The results of the sensitivity analyses can be found in Additional file 4.

## Discussion

Results of this study showed that a HE + PA intervention had a higher likelihood of being cost-effective compared with usual care among women at increased risk of GDM than a HE only or PA only intervention. At 35–37 weeks, the HE + PA intervention was significantly more effective than usual care in preventing gestational weight gain, whereas the HE and PA interventions were not. There was no significant beneficial effect on fasting glucose, HOMA-IR, and QALYs. At 35–37 weeks, the HE + PA intervention’s cost-effectiveness for gestational weight gain depends on the decision-makers’ willingness to pay per kilogram reduction in gestational weight gain, whereas the intervention does not seem to be cost-effective for fasting glucose and HOMA-IR. The latter is due to the fact that the maximum probability of cost-effectiveness was relatively low for fasting glucose (i.e. 0.70) and relatively high probabilities of cost-effectiveness (i.e. >0.90) are only reached for HOMA-IR of decision-makers are willing to pay large amounts of money per unit of effect gained (i.e. €25,000/unit). After the delivery of babies, the HE + PA intervention was cost-effective compared with usual care for QALYs. To illustrate, at the lower bounds of the Dutch and UK WTP-threshold for QALYs (i.e. €10,000 and €24,400/QALY gained, respectively), the probability of the HE + PA intervention being cost-effective compared with usual care was ≥0.93. Except for the complete-case analysis, results were supported by the sensitivity analyses. The difference in results between the main analysis and the complete-case analysis is likely due to selective drop-out. That is, differences were found between participants with complete and incomplete data, making the results of the imputed analysis more valid [41].

### Comparison with the literature

Only a few studies have evaluated the cost-effectiveness of lifestyle interventions for pregnant women at increased risk of GDM. Oostdam et al., for example, found a physical activity promotion intervention for pregnant women at increased risk of GDM not to be cost-effective compared with usual care for fasting glucose, insulin sensitivity, birth weight, and QALYs [42]. Although the results of the current study were slightly more positive, both studies did not show significant differences in societal costs and effects between a physical activity promotion intervention and usual care [42]. Kolu et al. did find a significant effect on birth weight, but did not find that a healthy eating and physical activity promotion intervention for women at increased risk of GDM was cost-effective compared with usual care for birth weight, QALYs, and perceived health. Kolu et al., however, included women with a BMI ≥ 25 kg/m^2^ instead of ≥29 kg/m^2^, which might explain why their healthy eating and physical activity intervention was not cost-effective in comparison with usual care for QALYs, whereas the present one was. Dodd et al. found a healthy eating and physical activity promotion intervention for pregnant women at increased risk of GDM not to be associated with significant cost savings, but with a high probability of cost-effectiveness for having an infant birth weight below 4 kg [43]. The latter intervention, however, did not have a significant impact on gestational weight gain. Recently, Poston et al. (2017) performed an economic evaluation in which they compared a physical activity and nutrition intervention for pregnant women at risk of GDM with usual care. From the NHS perspective, they found the intervention not to be cost-effective (i.e. at a WTP of £30,000/QALY its probability of cost-effectiveness was 0.01). This is in contrast with the present findings and might be explained by the fact that women were only followed up until 36 weeks of gestation, instead of until after delivery [44].

### Strengths and limitations

This study had several strengths, including its European multicenter randomized controlled trial design, its pragmatic trial design with a usual care control condition, its use of objectively measured clinical outcomes, its use of hospital records for collecting resource use data concerning the delivery of babies and other delivery-related in-hospital services (i.e. the biggest cost driver), as well as its use of state-of-the-art statistical methods, such as multiple imputation, bootstrapping, and multilevel analyses.

Several limitations are noteworthy as well. First, some resource use data were collected using self-report of participants, which may have caused “social desirability” and/or “recall bias”. Second, due to a limited availability of unit costs reflecting “true costs” for most of the participating countries we were not able to use multi-country unit cost data. To deal with this limitation, unit costs were based on Dutch costing data and were adjusted per country using purchasing power parities [45]. We do not expect this limitation to have greatly influenced our results, as the results were similar when using UK unit costs. Nonetheless, as the use of multi-country cost data is preferred in economic evaluations [46], the development of more country-specific costing manuals, including readily available unit costs, is encouraged. Third, a relatively large number of participants had some missing data. To deal with this limitation, missing data were multiply imputed, which is generally acknowledged as a more valid strategy for dealing with missing cost-effectiveness data than naïve methods, such as mean imputation [41].

### Implications for practice

The results of the present study indicate that after an initial investment in setting up the lifestyle coaching intervention and enrollment infrastructure, the HE + PA intervention was cost-effective compared with usual care for QALYs, which was mostly due to large reductions in costs related to the delivery of babies. In addition to being cost-effective for QALYs, this intervention also limited gestational weight gain, which is relevant for weight development of the women postpartum. As such, this study lends support for implementing a healthy eating and physical activity promotion intervention among pregnant women at increased risk of GDM broadly.

## Conclusion

A HE + PA intervention was found to have a higher likelihood of being cost-effective compared with usual care among women at increased risk of GDM than a HE only or PA only intervention. After 35–37 weeks, the HE + PA intervention’s cost-effectiveness for gestational weight gain depends on the decision-makers’ willingness to pay per kilogram reduction in gestational weight gain, whereas it was not cost-effective for fasting glucose and HOMA-IR. After the delivery of babies, the HE + PA intervention was cost-effective for QALYs, lending support for a broad implementation of a healthy eating and physical activity intervention among pregnant women at increased risk of GDM.

## Additional files

Additional file 1: CONSORT 2010 checklist of information to include when reporting a randomized trial. (DOC 217 kb)

Additional file 2: TIDieR (Template for Intervention Description and Replication) Checklist. (DOCX 39 kb)

Additional file 3: Unit costs used for valuing resource use in the main analysis. (DOCX 493 kb)

Additional file 4: Results of the sensitivity analyses. (DOCX 116 kb)
