# Supplementary material for: Cost-effectiveness of healthy eating and/or physical activity promotion in pregnant women at increased risk of gestational diabetes mellitus: economic evaluation alongside the DALI study, a European multicenter randomized controlled trial
Source: Int J Behav Nutr Phys Act. 2018 Mar 14;15:23. doi: 10.1186/s12966-018-0643-y (PMC5853142; doi:10.1186/s12966-018-0643-y)
Supplement: Supplementary file 2 — TIDieR (Template for Intervention Description and Replication) Checklist. (DOCX 39 kb) [file 12966_2018_643_MOESM2_ESM.docx]

**
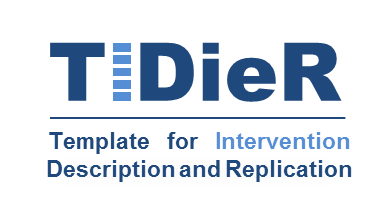
The TIDieR (Template for Intervention Description and Replication) Checklist*:**

Information to include when describing an intervention and the location of the information

| **Item number** | **Item** | **Where located **** | |
| --- | --- | --- | --- |
|  |  | Primary paper  (page or appendix  number) | Other ^†^ (details) |
|  | **BRIEF NAME** |  |  |
| **1.** | **Provide the name or a phrase that describes the intervention.** The DALI intervention is a healthy eating and/or physical activity promotion intervention targeted at pregnant women at increased risk of gestational diabetes mellitus. | ______9______ | ______________ |
|  | **WHY** |  |  |
| **2.** | **Describe any rationale, theory, or goal of the elements essential to the intervention.** The DALI intervention builds on principles of patient empowerment and cognitive behavioural techniques, inspired by Motivational Interviewing (MI). The overarching intervention framework was derived from a previous lifestyle trial aimed at diabetes prevention. | ____________ | Jelsma, Judith GM, et al. "DALI: Vitamin D and lifestyle intervention for gestational diabetes mellitus (GDM) prevention: an European multicentre, randomised trial–study protocol." *BMC pregnancy and childbirth* 13.1 (2013): 142. (page 4 of 16) |
|  | **WHAT** |  |  |
| **3.** | **Materials: Describe any physical or informational materials used in the intervention, including those provided to participants or used in intervention delivery or in training of intervention providers. Provide information on where the materials can be accessed (e.g. online appendix, URL).**  The DALI intervention entails individual sessions with a lifestyle coach. To facilitate the coaches and promote treatment integrity, each coach will have a desk-file outlining the intervention and options in detail and will use a Personal Digital Assistant (PDA) to provide a framework for the visit and to help guide the coach to deliver the intervention. Action cards will be used as a tool to help women formulate a specific and realistic goal, involving questions to alter the chances of success: e.g., “What is your action plan?, When will you start?” Participants receive a toolkit with useful materials to help them change their behavior, including a participant manual, with general information about (risk of developing) gestational diabetes mellitus and weight management. | ____________ | Jelsma, Judith GM, et al. "DALI: Vitamin D and lifestyle intervention for gestational diabetes mellitus (GDM) prevention: an European multicentre, randomised trial–study protocol." *BMC pregnancy and childbirth* 13.1 (2013): 142. (page 4,5 of 16) |
| **4.** | **Procedures: Describe each of the procedures, activities, and/or processes used in the intervention, including any enabling or support activities.**  Lifestyle coaching was offered during five face-to-face sessions of 30–45 minutes and four optional telephone calls of ≤20 minutes that occurred between the face-to-face sessions. | _____9_______ | _____________ |
|  | **WHO PROVIDED** |  |  |
| **5.** | **For each category of intervention provider (e.g. psychologist, nursing assistant), describe their expertise, background and any specific training given.** The appointed lifestyle coaches have been selected for their natural ability to be emphatic, and most had a background in either behavioural change, healthy eating and/or physical activity. They received a special training program containing  MI techniques to help women overcome their ambivalence or barriers that keep them from making the desired lifestyle changes. | ____________ | Jelsma, Judith GM, et al. "DALI: Vitamin D and lifestyle intervention for gestational diabetes mellitus (GDM) prevention: an European multicentre, randomised trial–study protocol." *BMC pregnancy and childbirth* 13.1 (2013): 142. (page 6 of 16) |
|  | **HOW** |  |  |
| **6.** | **Describe the modes of delivery (e.g. face-to-face or by some other mechanism, such as internet or telephone) of the intervention and whether it was provided individually or in a group.**  Lifestyle coaching was offered during five face-to-face sessions and four optional telephone calls that occurred between the face-to-face sessions. The intervention was delivered at an individual basis. | ______9______ |  |
|  | **WHERE** |  |  |
| **7.** | **Describe the type(s) of location(s) where the intervention occurred, including any necessary infrastructure or relevant features.** The one-to-one sessions took place either in the home of the participants or in the hospital/midwife practice/general practice, depending on local arrangements. | ______9______ | _____________ |
|  | **WHEN and HOW MUCH** |  |  |
| **8.** | **Describe the number of times the intervention was delivered and over what period of time including the number of sessions, their schedule, and their duration, intensity or dose.**  Lifestyle coaching was offered during five face-to-face sessions of 30–45 minutes and four optional telephone calls of ≤20 minutes that occurred between the face-to-face sessions. | _____________ | Jelsma, Judith GM, et al. "Is a motivational interviewing based lifestyle intervention for obese pregnant women across Europe implemented as planned? Process evaluation of the DALI study." Under review *BMC pregnancy and childbirth* |
|  | **TAILORING** |  |  |
| **9.** | **If the intervention was planned to be personalised, titrated or adapted, then describe what, why, when, and how.** It is anticipated that barriers to change may shift throughout the pregnancy  period, demanding continued tailored support to keep women on track with managing their gestational weight gain, healthy eating and physical activity. The timing of  the telephone contacts and the time between these contacts is dependent on the preference of the participant and the availability of the lifestyle coach, in order to deliver the intervention with the most appropriate timing and to  optimize chances of the uptake of behaviour change.  To prepare consultations, answers from the baseline questionnaire on the ‘ readiness to change scores’  for all the physical activity and healthy eating messages will be  loaded on the PDA and can be used in helping to tailor the intervention, e.g. explain risk factors and identify barriers to change. Participants will choose one or more  key items (agenda setting) as their main area of concern: risk factors of GDM, weight management, healthy eating and/or physical activity. The last two can only be chosen  once subjects are allocated to these intervention groups. Once a message has been selected, the first phase of behaviour change is centred on intention formation (motivation). Since an individual’ s readiness to change is variable and dynamic, these  scales can be used in follow up consultations as well to gain insight in the development of the women’ s changes she already experienced and serve as a feedback tool. | _____________ | Jelsma, Judith GM, et al. "DALI: Vitamin D and lifestyle intervention for gestational diabetes mellitus (GDM) prevention: an European multicentre, randomised trial–study protocol." *BMC pregnancy and childbirth* 13.1 (2013): 142. (page 4,5 of 16) |
|  | **MODIFICATIONS** |  |  |
| **10.^ǂ^** | Applicable?? | _____________ | _____________ |
|  | **HOW WELL** |  |  |
| **11.** | **Planned: If intervention adherence or fidelity was assessed, describe how and by whom, and if any strategies were used to maintain or improve fidelity, describe them.** Data on reach, dose delivered, fidelity and satisfaction were collected by independent researchers. Weekly recruitment reports, notes from meetings, coaching logs and evaluation questionnaires were consulted. Fidelity of lifestyle coaches was assessed by analysing audio recorded counselling sessions using the MI treatment integrity scale.   The DALI coaches received in total 32 hours of training in order to improve fidelity. At the beginning of the DALI study, a 2-day central training was offered to the coaches, led by experienced MI trainers and included role-play exercises and video recordings. Coaches received individualized feedback on their performance. A second 2-day training was delivered a few months later to review the coaches’ MI competency, share experiences and receive feedback. | _____________ | Jelsma, Judith GM, et al. "Is a motivational interviewing based lifestyle intervention for obese pregnant women across Europe implemented as planned? Process evaluation of the DALI study." Under review *BMC pregnancy and childbirth* |
| **12.^ǂ^** | **Actual: If intervention adherence or fidelity was assessed, describe the extent to which the intervention was delivered as planned.**  On average, 4 of the 5 face-to-face sessions were delivered. Mean fidelity to MI techniques almost reached expert opinion threshold for its global scores, but was below beginning proficiency for behavioral counts. No significant associations were found between process elements and gestational weight gain. | _____________ | Jelsma, Judith GM, et al. "Is a motivational interviewing based lifestyle intervention for obese pregnant women across Europe implemented as planned? Process evaluation of the DALI study." Under review *BMC pregnancy and childbirth* |

** **Authors** - use N/A if an item is not applicable for the intervention being described. **Reviewers** – use ‘?’ if information about the element is not reported/not sufficiently reported.

† If the information is not provided in the primary paper, give details of where this information is available. This may include locations such as a published protocol or other published papers (provide citation details) or a website (provide the URL).

ǂ If completing the TIDieR checklist for a protocol, these items are not relevant to the protocol and cannot be described until the study is complete.

* We strongly recommend using this checklist in conjunction with the TIDieR guide (see *BMJ* 2014;348:g1687) which contains an explanation and elaboration for each item.

* The focus of TIDieR is on reporting details of the intervention elements (and where relevant, comparison elements) of a study. Other elements and methodological features of studies are covered by other reporting statements and checklists and have not been duplicated as part of the TIDieR checklist. When a **randomised trial** is being reported, the TIDieR checklist should be used in conjunction with the CONSORT statement (see [www.consort-statement.org](http://www.consort-statement.org)) as an extension of **Item 5 of the CONSORT 2010 Statement.** When a **clinical trial** **protocol** is being reported, the TIDieR checklist should be used in conjunction with the SPIRIT statement as an extension of **Item 11 of the SPIRIT 2013 Statement** (see [www.spirit-statement.org](http://www.spirit-statement.org)). For alternate study designs, TIDieR can be used in conjunction with the appropriate checklist for that study design (see [www.equator-network.org](http://www.equator-network.org)).
