# Supplementary material for: Cost-effectiveness of healthy eating and/or physical activity promotion in pregnant women at increased risk of gestational diabetes mellitus: economic evaluation alongside the DALI study, a European multicenter randomized controlled trial
Source: Int J Behav Nutr Phys Act. 2018 Mar 14;15:23. doi: 10.1186/s12966-018-0643-y (PMC5853142; doi:10.1186/s12966-018-0643-y)
Supplement: Supplementary file 3 — Unit costs used for valuing resource use in the main analysis. (DOCX 493 kb) [file 12966_2018_643_MOESM3_ESM.docx]

***Additional file 1: Unit costs used for valuing resource use in the main analysis***
