# Supplementary material for: Cost-effectiveness of healthy eating and/or physical activity promotion in pregnant women at increased risk of gestational diabetes mellitus: economic evaluation alongside the DALI study, a European multicenter randomized controlled trial
Source: Int J Behav Nutr Phys Act. 2018 Mar 14;15:23. doi: 10.1186/s12966-018-0643-y (PMC5853142; doi:10.1186/s12966-018-0643-y)
Supplement: Supplementary file 4 — Results of the sensitivity analyses. (DOCX 116 kb) [file 12966_2018_643_MOESM4_ESM.docx]

***Additional file 2: Results of the sensitivity analyses***

| **Sensitivity analysis 1 – QALY tariff** | | | | | | | | | |
| --- | --- | --- | --- | --- | --- | --- | --- | --- | --- |
| **HE + PA** | | | | | | | | | |
| **Outcome measure** | **Sample size** | | **∆C(95%CI)** | **∆E(95%CI)** | **ICER** | **Distribution CE-plane (%)** | | | |
|  | **Intervention** | **Control** | **€** | **Points** | **€/point** | **NE** | **SE** | **SW** | **NW** |
| QALYs | 107 | 104 | -1627 (-4000 to 556) | 0.02 (0.00 to 0.05) | -75149 | 7.6 | 88.7 | 3.4 | 0.3 |
| **HE** | | | | | | | | | |
| **Outcome measure** | **Sample size** | | **∆C(95%CI)** | **∆E(95%CI)** | **ICER** | **Distribution CE-plane (%)** | | | |
|  | **Intervention** | **Control** | **€** | **Points** | **€/point** | **NE** | **SE** | **SW** | **NW** |
| QALYs | 114 | 104 | 653 (-1997 to 3343) | 0.01 (-0.02 to 0.02) | -1270604 | 32.1 | 16.3 | 16.4 | 35.1 |
| **PA** | | | | | | | | | |
| **Outcome measure** | **Sample size** | | **∆C(95%CI)** | **∆E(95%CI)** | **ICER** | **Distribution CE-plane (%)** | | | |
|  | **Intervention** | **Control** | **€** | **Points** | **€/point** | **NE** | **SE** | **SW** | **NW** |
| QALYs | 110 | 104 | -1155 (-3473 to 1142) | -0.01 (-0.03 to 0.02) | 120936 | 3.2 | 21.0 | 62.3 | 13.4 |
| **Sensitivity analysis 2 – UK unit costs** | | | | | | | | | |
| **HE + PA** | | | | | | | | | |
| **Outcome measure** | **Sample size** | | **∆C(95%CI)** | **∆E(95%CI)** | **ICER** | **Distribution CE-plane (%)** | | | |
|  | **Intervention** | **Control** | **€** | **Points** | **€/point** | **NE** | **SE** | **SW** | **NW** |
| Gestational weight gain | 107 | 104 | 421 (-536 to 1326) | -2.5 (-3.9 to 1.0) | -171 | 79.7 | 20.2 | 0.0 | 0.0 |
| Fasting glucose | 107 | 104 | 421 (-536 to 1326) | 0.0 (10.2 to 0.1) | -11567 | 56.3 | 14.9 | 5.4 | 23.4 |
| HOMA-IR | 107 | 104 | 421 (-536 to 1326) | 0.0 (-0.1 to 0.2) | 8854 | 54.0 | 12.4 | 7.9 | 25.7 |
| QALYs | 107 | 104 | -1160 (-3145 to 686) | 0.02 (0.00 to 0.04) | -67417 | 14.6 | 82.2 | 2.7 | 0.5 |
| **HE** | | | | | | | | | |
| **Outcome measure** | **Sample size** | | **∆C(95%CI)** | **∆E(95%CI)** | **ICER** | **Distribution CE-plane (%)** | | | |
|  | **Intervention** | **Control** | **€** | **Points** | **€/point** | **NE** | **SE** | **SW** | **NW** |
| Gestational weight gain | 114 | 104 | 701 (-251 to 1547) | -0.9 (-2.6 to 0.9) | -809 | 76.7 | 6.6 | 1.3 | 15.4 |
| Fasting glucose | 114 | 104 | 701 (-251 to 1547) | 0.1 (0.0 to 0.2) | 5744 | 2.3 | 0.3 | 7.6 | 89.8 |
| HOMA-IR | 114 | 104 | 701 (-251 to 1547) | 0.1 (-0.1 to 0.3) | 6449 | 79.7 | 6.9 | 1.0 | 12.4 |
| QALYs | 114 | 104 | 80 (-2041 to 2283) | 0.00 (-0.02 to 0.00) | -27825 | 17.4 | 20.6 | 26.9 | 35.1 |
| **PA** | | | | | | | | | |
| **Outcome measure** | **Sample size** | | **∆C(95%CI)** | **∆E(95%CI)** | **ICER** | **Distribution CE-plane (%)** | | | |
|  | **Intervention** | **Control** | **€** | **Points** | **€/point** | **NE** | **SE** | **SW** | **NW** |
| Gestational weight gain | 110 | 104 | 518 (-391 to 1392) | 0.1 (-1.5 to 1.7) | 6876 | 40.6 | 7.0 | 7.2 | 45.2 |
| Fasting glucose | 110 | 104 | 518 (-391 to 1392) | 0.0 (-0.1 to 0.1) | 220962) | 41.8 | 7.7 | 6.5 | 44.0 |
| HOMA-IR | 110 | 104 | 518 (-391 to 1392) | 0.1 (-0.1 to 0.2) | 9316 | 61.8 | 9.9 | 4.3 | 24.0 |
| QALYs | 110 | 104 | -947 (-2938 to 970) | 0.00 (-0.02 to 0.01) | 106545 | 2.7 | 15.1 | 65.8 | 16.4 |
| **Sensitivity analysis 3 – Healthcare perspective** | | | | | | | | | |
| **HE + PA** | | | | | | | | | |
| **Outcome measure** | **Sample size** | | **∆C(95%CI)** | **∆E(95%CI)** | **ICER** | **Distribution CE-plane (%)** | | | |
|  | **Intervention** | **Control** | **€** | **Points** | **€/point** | **NE** | **SE** | **SW** | **NW** |
| Gestational weight gain | 107 | 104 | 496 (292 to 740) | -2.3 (-3.7 to -0.9) | 215 | 100.0 | 0.0 | 0.0 | 0.0 |
| Fasting glucose | 107 | 104 | 496 (292 to 740) | 0.0 (-0.2 to 0.1) | -12002 | 73.1 | 0.0 | 0.0 | 26.9 |
| HOMA-IR | 107 | 104 | 496 (292 to 740) | 0.0 (-0.1 to 0.2) | 11705 | 63.7 | 0.0 | 0.0 | 36.3 |
| QALYs | 107 | 104 | -1063 (-2838 to 472) | 0.02 (0.00 to 0.04) | -59610 | 10.2 | 86.5 | 3.0 | 0.4 |
| **HE** | | | | | | | | | |
| **Outcome measure** | **Sample size** | | **∆C(95%CI)** | **∆E(95%CI)** | **ICER** | **Distribution CE-plane (%)** | | | |
|  | **Intervention** | **Control** | **€** | **Points** | **€/point** | **NE** | **SE** | **SW** | **NW** |
| Gestational weight gain | 114 | 104 | 364 (205 to 501) | -0.6 (-2.4 to 1.2) | -593 | 76.0 | 0.0 | 0.0 | 24.1 |
| Fasting glucose | 114 | 104 | 364 (205 to 501) | 0.1 (0.0 to 0.3) | 2945 | 3.5 | 0.0 | 0.0 | 96.5 |
| HOMA-IR | 114 | 104 | 364 (205 to 501) | 0.2 (0.0 to 0.3) | 2414 | 92.7 | 0.0 | 0.0 | 7.3 |
| QALYs | 114 | 104 | -85 (-2081 to 2005) | 0.00 (-0.02 to 0.02) | 31621 | 17.2 | 20.5 | 33.4 | 28.8 |
| **PA** | | | | | | | | | |
| **Outcome measure** | **Sample size** | | **∆C(95%CI)** | **∆E(95%CI)** | **ICER** | **Distribution CE-plane (%)** | | | |
|  | **Intervention** | **Control** | **€** | **Points** | **€/point** | **NE** | **SE** | **SW** | **NW** |
| Gestational weight gain | 110 | 104 | 345 (206 to 464) | 0.1 (-1.4 to 1.7) | 2342 | 42.0 | 0.0 | 0.0 | 58.0 |
| Fasting glucose | 110 | 104 | 345 (206 to 464) | 0.0 (-0.1 to 0.1) | -36265 | 57.7 | 0.0 | 0.0 | 42.3 |
| HOMA-IR | 110 | 104 | 345 (206 to 464) | 0.1 (-0.1 to 0.3) | 5498 | 72.3 | 0.0 | 0.0 | 27.7 |
| QALYs | 110 | 104 | -1872 (-3512 to -460) | 0.00 (-0.03 to 0.01) | 236938 | 0.0 | 19.2 | 80.1 | 0.5 |
| **Sensitivity analysis 4 – Complete case analysis** | | | | | | | | | |
| **HE + PA** | | | | | | | | | |
| **Outcome measure** | **Sample size** | | **∆C(95%CI)** | **∆E(95%CI)** | **ICER** | **Distribution CE-plane (%)** | | | |
|  | **Intervention** | **Control** | **€** | **Points** | **€/point** | **NE** | **SE** | **SW** | **NW** |
| Gestational weight gain | 107 | 104 | 710 (-819 to 2120) | -2.4 (-3.7 to -1.1) | -295 | 84.0 | 16.0 | 0.0 | 0.0 |
| Fasting glucose | 107 | 104 | 710 (-819 to 2120) | -0.1 (-0.3 to 0.0) | -6269 | 79.6 | 15.3 | 0.7 | 4.3 |
| HOMA-IR | 107 | 104 | 710 (-819 to 2120) | -0.1 (-0.2 to 0.11) | `-11789 | 20.6 | 2.6 | 13.5 | 6.3 |
| QALYs | 107 | 104 | 749 (-2039 to 3280) | 0.02 (-0.02 to 0.05) | 46782 | 61.1 | 23.4 | 4.0 | 11.5 |
| **HE** | | | | | | | | | |
| **Outcome measure** | **Sample size** | | **∆C(95%CI)** | **∆E(95%CI)** | **ICER** | **Distribution CE-plane (%)** | | | |
|  | **Intervention** | **Control** | **€** | **Points** | **€/point** | **NE** | **SE** | **SW** | **NW** |
| Gestational weight gain | 114 | 104 | 993 (-453 to 2292) | -1.0 (-2.5 to 0.4) | -936 | 84.5 | 7.4 | 0.0 | 0.0 |
| Fasting glucose | 114 | 104 | 993 (-453 to 2292) | 0.1 (0.0 to 0.2) | 12455 | 10.1 | 1.1 | 6.9 | 81.9 |
| HOMA-IR | 114 | 104 | 993 (-453 to 2292) | 0.0 (-0.2 to 0.1) | 2000346 | 45.1 | 3.0 | 4.9 | 46.9 |
| QALYs | 114 | 104 | 312 (-2297 to 2917) | -0.04 (-0.03 to 0.02) | -79247 | 22.6 | 14.7 | 26.0 | 36.6 |
| **PA** | | | | | | | | | |
| **Outcome measure** | **Sample size** | | **∆C(95%CI)** | **∆E(95%CI)** | **ICER** | **Distribution CE-plane (%)** | | | |
|  | **Intervention** | **Control** | **€** | **Points** | **€/point** | **NE** | **SE** | **SW** | **NW** |
| Gestational weight gain | 110 | 104 | 967 (-618 to 2346) | 0.3 (-1.1 to 1.8) | 3053 | 29.5 | 3.5 | 7.1 | 60.0 |
| Fasting glucose | 110 | 104 | 967 (-618 to 2346) | 0.0 (-0.1 to 0.1) | -91049 | 51.1 | 6.9 | 3.7 | 38.4 |
| HOMA-IR | 110 | 104 | 967 (-618 to 2346) | 0.1 (-0.1 to 0.3) | 11723 | 71.1 | 7.6 | 3.0 | 18.4 |
| QALYs | 110 | 104 | 313 (-2359 to 2651) | -0.01 (-0.04 to 0.02) | -22581 | 11.8 | 7.2 | 31.6 | 49.3 |

Abbreviations: C: Costs; E: Effects; ICER: Incremental Cost-Effectiveness Ratio; CE-plane: Cost-Effectiveness plane; NE: Northeast-Quadrant; SE: Southeast-Quadrant; NW; Northwest-Quadrant; ZW; Southwest-Quadrant; QALYs: Quality Adjusted Life Years; HOMA-IR: HOMA index – Insulin Resistance
